# Influence of Aging Precipitation Kinetics on Mechanical Properties and Corrosion Behavior of the Al-1.1Cu-2.4Li-X Alloy

**DOI:** 10.3390/ma19142984

**Published:** 2026-07-10

**Authors:** Danyang Liu, Hao Cheng, Huabo Gu, Jianmei Li, Chao Cai, Kefu Gan, Guangjun Zeng, Miao Song, Jinfeng Li

**Affiliations:** 1 School of Materials Science and Engineering, Central South University, Changsha 410083, Chinalijinfeng@csu.edu.cn (J.L.); 2 CSSC Qingdao Beihai Shipbuilding Co., Ltd., Qingdao 266520, China; 3 School of Chemistry and Chemical Engineering, Ningxia University, Yinchuan 750001, China

**Keywords:** Al–Li alloy, aging treatment, precipitates, corrosion behavior

## Abstract

The mechanical properties, intergranular corrosion, and exfoliation corrosion of T6- and T8-aged Al–1.1Cu–2.4Li–X alloys (high Li content, Cu/Li ratio ≈ 0.46) were systematically examined. Microstructural analysis shows that T8 aging produces a higher number density of T1 (Al2CuLi) precipitates and a higher number density, as well as a finer and more uniform distribution of δ′ (Al3Li) compared to T6 aging. This results in yield and tensile strengths that are 90 MPa and 40 MPa higher, respectively, for the T8 condition. In terms of corrosion, the T6-aged alloy displays intergranular corrosion depths of 176.5–210.1 μm, while the T8-aged alloy exhibits much shallower depths of 67.2–95.8 μm. The inferior resistance to intergranular and exfoliation corrosion in the T6 temper is attributed to precipitation-free zones (PFZs) and secondary-phase particles at grain boundaries. Increasing T6’s aging time reduces the PFZ width and thus improves corrosion resistance. Conversely, prolonged T8 aging causes a gradual decrease in corrosion potential and an increase in corrosion current density, linked to a higher density of T1 precipitates, which signifies increased corrosion susceptibility. These findings provide a reference for understanding corrosion mechanisms and improving the corrosion resistance of high-Li Al–Li alloys.

## 1. Introduction

Al–Li alloys are used as lightweight structural materials in the aerospace field due to their excellent properties, including high strength and great corrosion properties [1,2]. Among these, Al–Li alloys with a high Li content (Li content ≥ 2%) exhibit outstanding characteristics such as a low density, high elastic modulus, excellent cryogenic performance, and plasticity, making them suitable for use in fuel tanks for storing cryogenic propellants such as liquid hydrogen [3,4]. However, a high Li content also leads to some issues, including insufficient ductility, anisotropy, and poor corrosion resistance, which severely restrict their wider engineering applications in the aerospace sector. Thus, controlling the microstructure of high-Li-content Al–Li alloys by adjusting heat treatment processes to enhance their corrosion resistance is of significant practical importance for industrial applications [5].

The main corrosion behaviors of Al–Li alloys include intergranular corrosion, exfoliation corrosion, and stress corrosion cracking [6,7,8]. The corrosion behaviors are closely related to the grain structure and precipitation behavior. Existing research indicates that the role of Li-rich phases (such as Al_3_Li precipitates) in pitting corrosion is not unidirectional; δ′ (Al_3_Li) can act as either an anode or a cathode depending on the matrix chemistry and aging condition [9,10]. During aging, the primary strengthening phase (T_1_ precipitates) in Al–Li alloys gradually nucleates within the grains, significantly reducing the Cu content in the matrix and causing its potential to shift toward the negative [11,12,13]. This further reduces the driving force for intergranular corrosion, causing the primary corrosion mechanism to transition from intergranular corrosion to pitting corrosion [14]. Exfoliation corrosion propagates extensively into the material through grain boundary pathways, leading to delamination and exfoliation of the surface metal layer. In high-Li-content Al–Li alloys, the susceptibility to exfoliation corrosion is governed largely by the continuity of Li-rich grain-boundary precipitates and the PFZ (precipitation-free zone) [15]. In accordance with depletion theory [16], the corrosion potential of the PFZ is lower than that of the secondary-phase particles, thereby causing the PFZ band to be preferentially dissolved as the anode. Recent studies reveal that trace Zn addition promotes the incorporation of Zn into coarse-grained precipitation phases at grain boundaries, effectively reducing the potential difference between these phases and the surrounding matrix. This inhibits intergranular and exfoliation corrosion while maintaining the alloy’s strength [17]. In summary, the corrosion behaviors of Al–Li alloys are closely related to the type of precipitates, their electrochemical characteristics, and the microstructure of the grain boundaries. This provides a crucial basis for the synergistic regulation of corrosion resistance and strength through the optimization of aging regimens.

Additionally, the aging regimens for Al–Li alloys include T3 aging (pre-deformation + natural aging), T4 aging (natural aging), T6 aging (artificial aging), and T8 aging (pre-deformation + artificial aging), in which T6 and T8 are the primary industrial aging practices [18]. During T6 and T8 aging, the precipitation sequence of Al–Li alloys is related to the Cu/Li ratio. Recent studies have shown that when the Cu/Li ratio ranges from 1.0 to 2.5, the precipitation sequence is SSSS→GP zone + δ′→θ′(Al_2_Cu) + δ′→δ′ + T_1_→T_1_; when the Cu/Li ratio decreases to <1.0, the precipitation sequence is SSSS→δ′ + T_1_→T_1_ [19]. Meanwhile, in contrast to δ′ precipitates, T_1_ precipitates exhibit the strongest strengthening effect, attributable to their formation on the {111}Al habit plane, which effectively impedes dislocation motion and thereby enhances the strength of Al–Li alloys [20]. For high-Li Al–Li alloys, strengthening of the alloy primarily relies on the uniform precipitation of the δ′ precipitates and a small amount of the θ′ precipitates during T6 aging, whereas T8 aging uses pre-deformation-induced dislocations as nucleation sites for the T_1_ precipitates, promoting the formation of fine T_1_ precipitates as the primary strengthening phase while effectively suppressing the coarsening of the δ′ precipitates [21,22,23,24]. Proton et al. [25] found that artificial aging promotes the formation of intergranular precipitates and an intragranular T_1_ phase in 2050 Al–Li alloy, causing the corrosion mode to change from intergranular corrosion to intragranular corrosion and significantly improving the corrosion resistance of the alloy. Jiang et al. [26] mentioned that when 2197 Al–Li alloy is aged at 160 °C, the T_1_ precipitates exhibit a uniform distribution and fine size, and the corrosion resistance of the alloy is optimized. However, excessively long aging leads to coarsening of the T_1_ precipitates and widening of the PFZ, which greatly increase the susceptibility to intergranular and exfoliation corrosion. Currently, research on the corrosion properties of Al–Li alloys mainly focuses on alloy systems in which the T_1_ precipitates serve as the primary precipitation strengthening phase [27,28,29], whereas the corrosion mechanisms of the high-Li-content Al–Li alloys with δ′ precipitates remain insufficiently explored. Furthermore, the influence of precipitation evolution on corrosion during T6 and T8 aging in high-Li-content Al–Li alloys requires further investigation.

This study systematically compares the corrosion properties of high-Li-content Al–Li alloys during T6 and T8 aging, establishes the intrinsic relationship between precipitate evolution and corrosion behavior, and elucidates the regulation mechanism of their corrosion resistance under these aging regimes. The findings may provide a reference for investigating corrosion mechanisms and enhancing the corrosion resistance of high-Li-content Al–Li alloys.

## 2. Materials and Experimental Details

**Figure 1:**
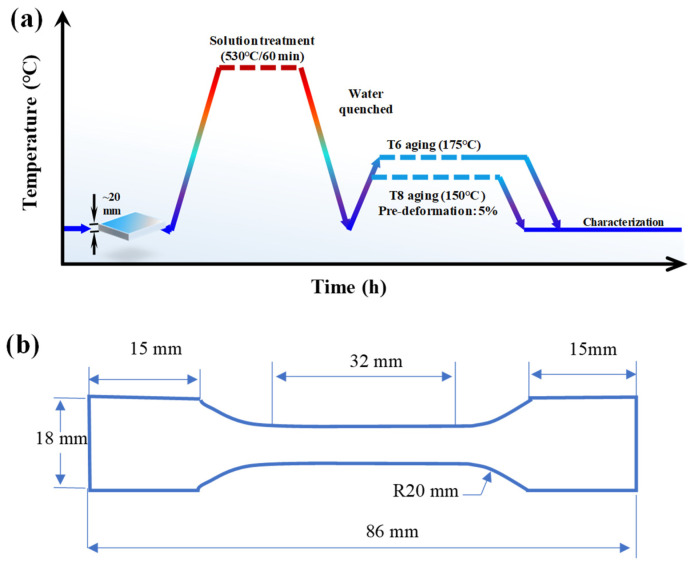
Schematic illustrations of (**a**) the heat treatment procedure and (**b**) the tensile test specimen configuration for the experimental alloys [3].

This study prepared alloys using high-purity aluminum (99.99 wt.%), lithium (99.99 wt.%), magnesium (99.99 wt.%), zinc (99.99 wt.%), Al-50 wt.% Mn, and Al-30 wt.% Zr master alloys provided by Zhuzhou Smelter Group Co., Ltd., Zhuzhou, China, and the chemical compositions of the experimental alloy are shown in Table 1. The ingots are first subjected to homogenization treatment, then hot-rolled to 20 mm thickness. The hot-rolled plate undergoes solution treatment at 530 °C for 1 h and is immediately water-quenched. Thereafter, selected samples are subjected to a T8 temper, consisting of 5% pre-deformation followed by aging at 150 °C. The deformation process is carried out at room temperature via a single pass through a rolling mill, ensuring that the alloy remains free from cracks whilst achieving 5% pre-deformation, and with the T8 aging times ranging from 4 h to 168 h. The other samples are subjected to T6 aging (175 °C), with T6 aging times ranging from 4 h to 168 h. In the above processes, the alloy preparation is carried out under an argon atmosphere, whilst all other heat treatments are carried out in air. The schematic diagram of the heat treatment process is shown in Figure 1 [3].

Tensile specimens with various aging treatments are prepared according to the Ref. [30], as shown in Figure 1b. For each aging treatment condition, the tensile specimens are tested three times. Mechanical properties measurements are conducted on an MTS-810 fatigue testing machine (MTS Systems Corporation, Eden Prairie, MN, USA) at a 2 mm/min strain rate. The intergranular corrosion immersion tests are performed according to the Ref. [31]. The solution used for intergranular corrosion testing is composed of 57 g/L NaCl and 10 mL/L H_2_O_2_ provided by Bolinda company, Shenzhen, China. The T6-aged and T8-aged alloy specimens are sealed with epoxy resin and subsequently immersed in this solution at 35 ± 2 °C for 6 h. After immersion, the surfaces are rinsed with distilled water and alcohol. The longitudinal cross-section surfaces of the corroded alloys are ground and polished for observation under an optical microscope (OM). To ensure the reliability of the intergranular corrosion results, three specimens are tested for each of the T6-aged and T8-aged alloys. According to Ref. [31], intergranular corrosion severity is classified into five grades: Grade 1: Corrosion depth ≤ 10 μm; Grade 2: 10~30 μm; Grade 3: 30~100 μm; Grade 4: 100~300 μm; Grade 5: Corrosion depth ≥ 300 μm.

The exfoliation corrosion test is conducted according to Ref. [32]. The corrosion solution contains 4.0 mol/L NaCl, 0.5 mol/L KNO_3_, and 0.1 mol/L HNO_3_ provided by Bolinda company, Shenzhen, China, with a pH of approximately 0.4. Exfoliation corrosion test specimens, with dimensions of 100 mm (length) × 50 mm (width) × 9 mm (thickness), are machined from alloys in the T6-aged and T8-aged conditions. The specimens are immersed in the corrosion solution for periods ranging from 4 h to 72 h. Subsequently, the corrosion specimens are visually inspected to evaluate the extent of exfoliation corrosion. According to the Ref. [32], exfoliation corrosion is classified into 8 grades, as follows: N: no obvious surface corrosion; PA: slight pitting on the surface; PB: moderate pitting on the surface; PC: severe pitting on the surface, with blistering; EA: localized blistering or slight delamination on the surface; EB: localized blistering or distinct delamination on the surface; EC: blistering on the surface with severe delamination, corrosion penetrating deep into the metal, and specimen thickening; ED: severe delamination penetrating deep into the metal, resulting in marked specimen thickening.

The electrochemical performance is tested using a three-electrode system comprising a reference electrode, a counter electrode, and a working electrode. The system is immersed in a 0.6 wt.% NaCl deionized water solution. The polarization curves and OCP (open-circuit potentials) of the T6-aged and T8-aged alloys are measured using a CHI660B electrochemical workstation (Chenhua Instrument company, Shanghai, China).

The secondary particles of the as-cast and homogenized samples are investigated using a FEI Quanta 200 SEM (scanning electron microscope, Thermo Fisher Scientific, Hillsboro, OR, USA) equipped with a 20 kV energy-dispersive X-ray spectrometer (EDS, Energy Dispersive Spectroscopy, Bruker company, Berlin, Germany). The samples for EPMA (Electron Probe Microanalysis) are prepared by an argon ion cross-section polisher (IB-19510CP, Gatan company, Pleasanton, CA, USA) to ensure the image quality and accuracy of elemental analysis. Elemental analysis is performed on a JXA-8530F electron probe (JEOL company, Tokyo, Japan).

XRD (X-ray diffraction) analysis is performed at a scanning rate of 0.01°/min. The evolution of the aging precipitates is characterized by TEM (transmission electron microscopy, Thermo Fisher Scientific, OR, USA) and HAADF-STEM (high-angle annular dark-field scanning transmission electron microscopy, Thermo Fisher Scientific, OR, USA). The TEM, HAADF-STEM, and EDS observations are performed using an FEI Tecnai F20 instrument (Thermo Fisher Scientific, OR, USA) at 200 kV. The TEM specimens are prepared using a dual-jet electrolytic polishing method with an electrolyte solution of 75 vol% CH_3_OH and 25 vol% HNO_3_ at temperatures ranging from −25 °C to −35 °C. Both sample preparation and sample soaking are carried out at room temperature.

## 3. Experimental and Characterization Results

### 3.1. Secondary-Phase Particle Distribution

**Figure 2:**
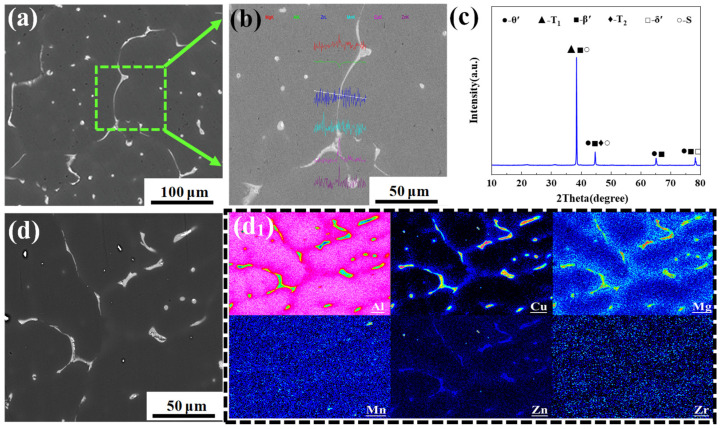
SEM image, XRD pattern, and EPMA results of the as-cast alloy. (**a**,**b**) SEM and line scanning images; (**c**) XRD pattern; (**d**,**d_1_**) EPMA results.

Figure 2 shows the SEM and EPMA images of the distribution of secondary-phase particles in the as-cast Al-1.1Cu-2.4Li-X alloy. As shown in Figure 2a,b, significant elemental segregation is observed at grain boundaries, forming a continuous network of coarse secondary-phase particles. Moreover, the coarse secondary-phase particles located at grain boundaries exhibit predominant enrichment in Cu, Mg, Mn, Zn, and Zr, and the insoluble secondary-phase particles within the grains are enriched in Cu and Mg elements, as shown in Figure 2(d,d_1_).

**Figure 3:**
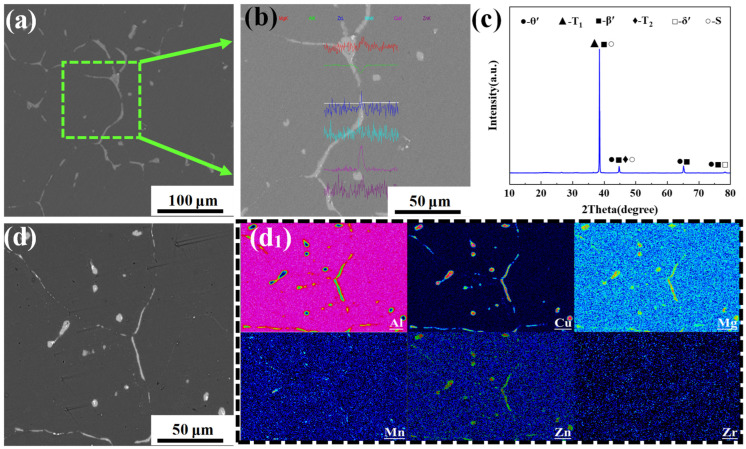
SEM image, XRD pattern, and EPMA results of the homogenized alloy. (**a**,**b**) SEM and line scanning images; (**c**) XRD pattern; (**d**,**d_1_**) EPMA results.

Figure 3 illustrates the SEM and EPMA images of the distribution of secondary-phase particles in the Al-1.1Cu-2.4Li-X alloy treated with homogenization heat treatment. As shown in Figure 3a,d, the dendritic structure is significantly reduced, the number density of coarse secondary-phase particles is markedly decreased, and the size of the residual eutectic phase is reduced. Nevertheless, the Cu-rich and Mg-rich phases are largely dissolved, with only a small number of discontinuously distributed and smaller-sized secondary-phase particles remaining in the matrix. Compared to the Al-1.1Cu-2.4Li-X alloy ingots, the area fraction of coarse secondary-phase particles is significantly decreased after homogenization heat treatment. However, some high-solubility Cu-rich phases and insoluble Mn-containing phases still remain at the grain boundaries and within the matrix, as shown in Figure 3(d,d_1_).

### 3.2. Mechanical Properties

**Figure 4:**
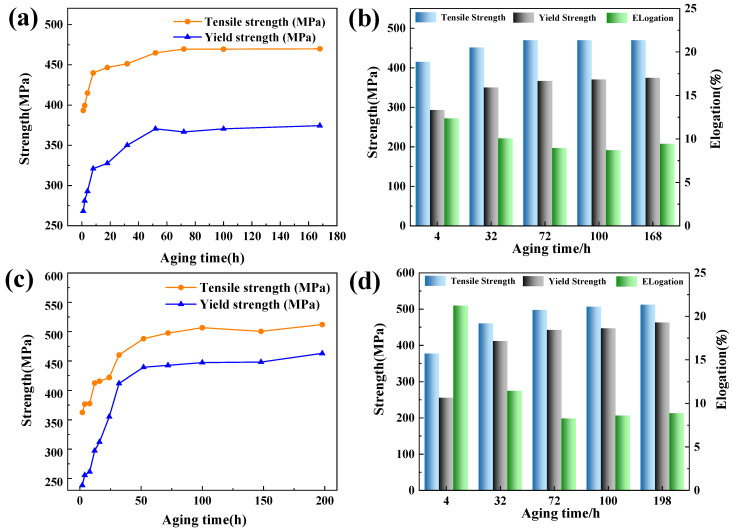
Mechanical properties of the alloys subjected to T6 and T8 aging treatments. (**a**,**b**) T6-aged condition; (**c**,**d**) T8-aged condition.

Figure 4 presents the strength and elongation of the T6-aged and T8-aged Al–1.1Cu–2.4Li–X alloy, with the corresponding yield strength, tensile strength, and elongation values tabulated in Table 2. As shown in Figure 4, the yield strength and tensile strength gradually increase with the prolongation of the T6 and T8 aging times. As shown in Table 2, the yield strength and tensile strength of the T6-4 h-aged alloy are 292.8 MPa and 414.9 MPa; the T6-168 h-aged specimen achieves the maximum yield strength and tensile strength of 374.4 MPa and 469.9 MPa, with an elongation of 9.4%. For T8 aging treatment, the yield strength and tensile strength of the T8-4 h-aged specimen are 255.6 MPa and 377.0 MPa, and the T8-168 h-aged specimen achieves a maximum yield strength and tensile strength of 463.0 MPa and 512.0 MPa, with an elongation of 8.9%. The tensile results indicate that the yield strength and tensile strength of the T8-aged alloy are higher than approximately 90 MPa and 40 MPa compared to those of the T6-aged alloy, while the elongation remains essentially the same.

### 3.3. Intergranular Corrosion

**Figure 5:**
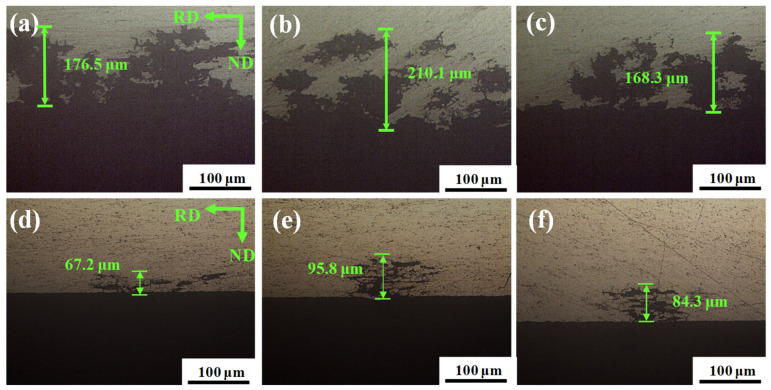
Cross-sectional images of intergranular corrosion tests in T6-aged and T8-aged alloys. (**a**–**c**) T6 aging; (**d**–**f**) T8 aging.

Figure 5 shows the typical corrosion morphologies and corresponding corrosion depths of T6-aged and T8-aged Al-1.1Cu-2.4Li-X alloys. After immersion tests for intergranular corrosion, the Al-1.1Cu-2.4Li-X alloy exhibits the following corrosion types (Table 3): Generalized intergranular corrosion: areas exhibiting a grid-like corrosion morphology (intergranular corrosion) accounted for more than 80% of the observed corrosion area. Localized intergranular corrosion: a grid-like corrosion morphology is present but covers less than 80% of the observed area. Pitting corrosion: minute corrosion pits appear in localized surface regions, and cross-sectional examination reveals pits of varying sizes. Pitting corrosion accompanied by intergranular corrosion: slight intergranular corrosion appears around the base of the corrosion pits. Typically, the depth of the re-emerging minor corrosion cracks is very small, whereas the resulting corrosion pits are generally larger.

As shown in Figure 5, the main corrosion types include general intergranular corrosion and pitting corrosion accompanied by intergranular corrosion. As illustrated in Figure 5a,c, the dominant corrosion mode in the T6-aged alloy is general intergranular corrosion, and the depth of intergranular corrosion ranges from 176.5 μm to 210.1 μm. As shown in Figure 5d,f, the T8-aged alloy exhibits reduced intergranular corrosion susceptibility, and the main corrosion type is pitting corrosion accompanied by intergranular corrosion, with intergranular corrosion depths ranging from 67.2 to 95.8 μm. These data reveal that, compared with the T8-aged alloy, the T6-aged alloy possesses greater intergranular corrosion susceptibility.

### 3.4. Exfoliation Corrosion

**Figure 6:**
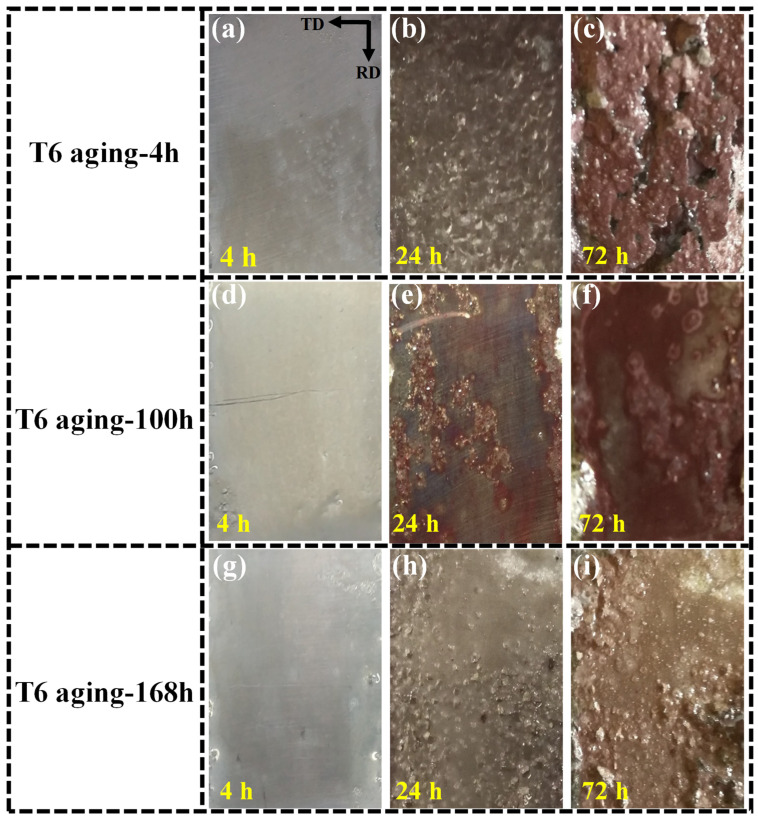
Macro-morphology images after exfoliation corrosion of T6-aged alloy. (**a**–**c**) T6 aging-4 h; (**d**–**f**) T6 aging-100 h; (**g**–**i**) T6 aging-168 h.

Figure 6 presents the surface morphology of the T6-aged Al-1.1Cu-2.4Li-X alloy after EXCO (exfoliation corrosion) testing, and the corresponding exfoliation corrosion levels are presented in Table 4. After 4 h of immersion, the surface morphology of the specimens subjected to various T6 aging treatments (4 h~168 h) shows no obvious corrosion signs, and the exfoliation corrosion levels are N. As the immersion time is extended to 24 h and 72 h, the exfoliation corrosion severity of the Al-1.1Cu-2.4Li-X alloy increases. After 24 h of immersion, relatively severe pitting corrosion appears on the surface of the T6-4 h-aged alloy, and the exfoliation corrosion level reaches PB. As the immersion time is further extended to 72 h, the number of localized blisters on the alloy surface increases, the blistered area expands, and surface delamination becomes severe, leading to a transition of the exfoliation corrosion level from PB to ED. Moreover, as the T6 aging time is extended to 100 h and 168 h, the corrosion level on the specimen surfaces decreases, indicating improved exfoliation corrosion resistance.

**Figure 7:**
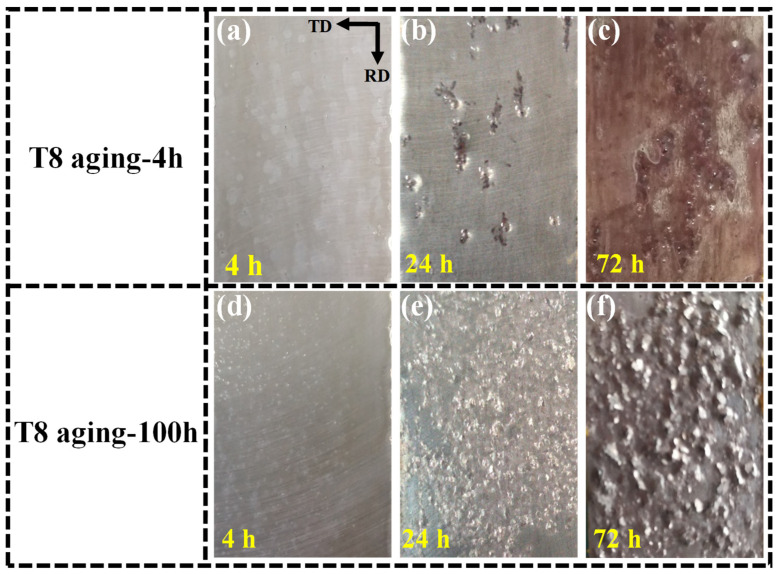
Macro-morphology images after exfoliation corrosion of T8-aged alloy. (**a**–**c**) T8 aging-4 h; (**d**–**f**) T8 aging-100 h.

Figure 7 illustrates the surface morphology of the T8-aged Al-1.1Cu-2.4Li-X alloy after exfoliation corrosion testing, and the corresponding exfoliation corrosion levels are presented in Table 4. After 4 h of immersion, the surface morphologies of the specimens subjected to various T8 aging treatments (4 h~100 h) show no obvious corrosion signs, and the exfoliation corrosion level is N. With increasing immersion time to 24 h and 72 h, the corrosion degree of the Al-1.1Cu-2.4Li-X alloy becomes more severe. After 24 h of immersion, minor pitting corrosion appears on the surface of the T8-4 h-aged alloy, and the exfoliation corrosion level reaches PA. As the immersion time further extends to 72 h, severe pitting corrosion occurs on the surface, and the exfoliation corrosion level changes from PA to PC. Furthermore, as the T8 aging time extends to 100 h, the corrosion level on the surface is higher, indicating reduced exfoliation corrosion resistance. Compared to the T6-aged alloy, the T8-aged alloy shows improved exfoliation corrosion resistance.

### 3.5. Precipitation Behavior

**Figure 8:**
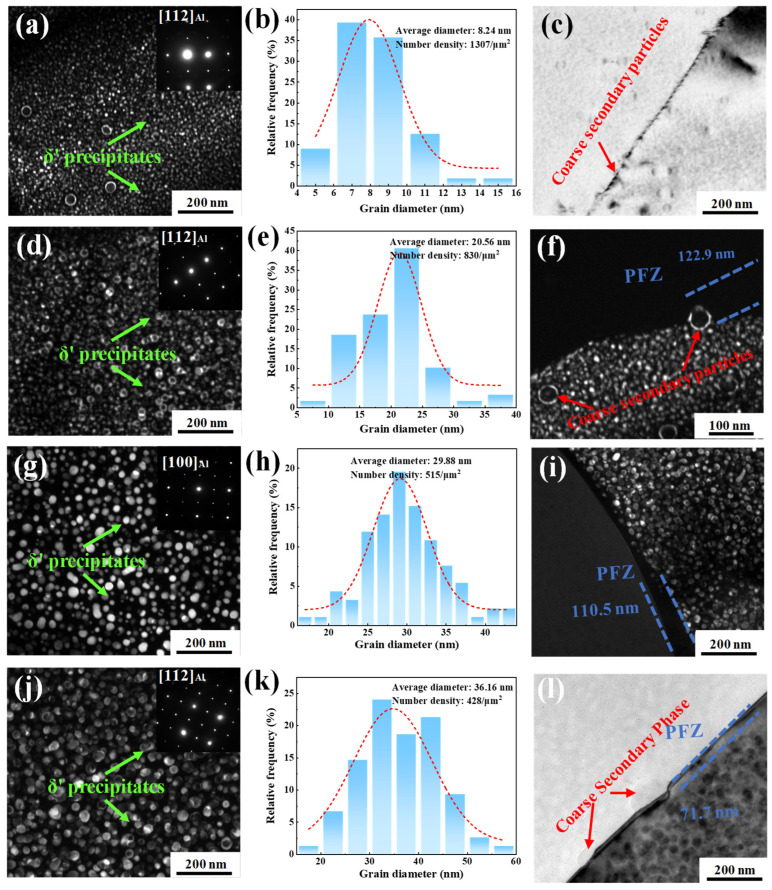
TEM images, SAED patterns, and the δ’ precipitate diameter distribution histograms of the T6-aged alloys. (**a**–**c**) T6 aging-4 h; (**d**–**f**) T6 aging-18 h; (**g**–**i**) T6 aging-100 h; (**j**–**l**) T6 aging-168 h.

Figure 8 displays the TEM micrographs, SAED (selected area electron diffraction) patterns, and histograms of precipitate diameter distribution for the T6-aged specimen. As shown in Figure 8a–c, spherical δ′ precipitates are clearly discernible in the T6-aged specimen, with a number density of 1307/μm^2^ and an average size of 8.2 nm. Meanwhile, coarse secondary-phase particles are distributed along grain boundaries in the T6-aged specimen. As shown in Figure 8d–l, when the T6 aging time is extended to 168 h, the δ′ precipitates gradually coarsen, their average size increases to 36.2 nm, and the number density decreases to 428/μm^2^. Meanwhile, the precipitation-free zone (PFZ) width of the T6-aged specimen gradually decreases from 122.9 nm to 71.7 nm.

**Figure 9:**
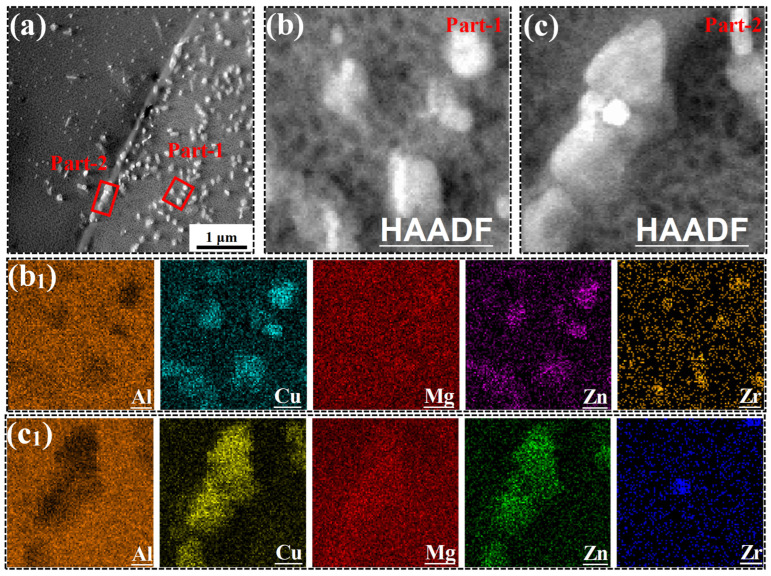
TEM images, HAADF-TEM images, and EDS mappings of the samples treated with T6 aging/100 h. (**a**) TEM image; (**b**,**c**) HAADF -TEM images; (**b_1_**,**c_1_**) EDS mappings.

Figure 9 shows the HAADF-STEM image and EDS-mapping results of the T6-aged Al-1.1Cu-2.4Li-X alloy. As shown in Figure 9b, dispersed secondary-phase particles are present within the grains and at the grain boundaries in the T6-aged alloy. EDS elemental mapping reveals that the intragranular secondary-phase particles are predominantly Cu-rich, Zn-rich, and Zr-rich phases, whereas those located at the grain boundaries consist primarily of Cu-rich and Zn-rich phases. These insoluble secondary-phase particles distributed at the grain boundaries and within the grains are retained from the homogenization treatment (Figure 3), which cannot dissolve during the subsequent solution treatment.

**Figure 10:**
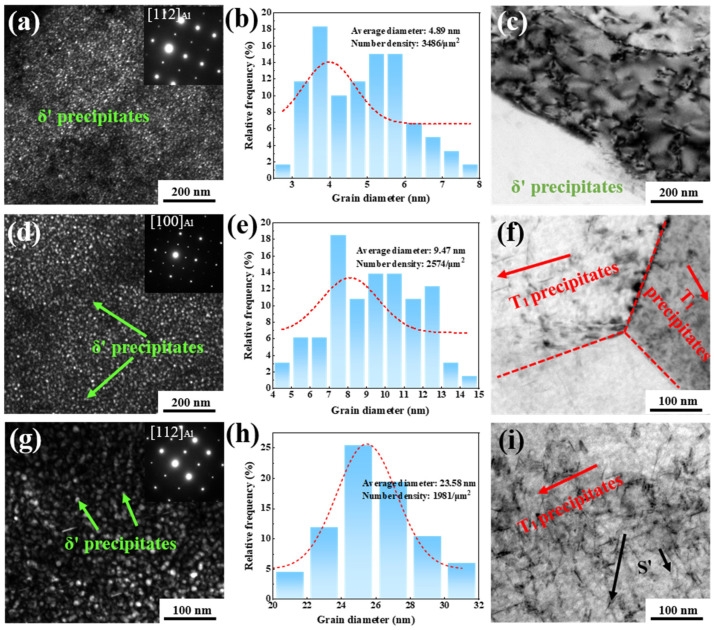
TEM images, SAED patterns, and the δ’ precipitate diameter distribution histograms of the T8-aged alloys. (**a**–**c**) T8 aging-4 h; (**d**–**f**) T8 aging-18 h; (**g**–**i**) T8 aging-100 h.

Figure 10 shows the TEM images, SAED patterns, and precipitate size distribution histograms of the T8-aged specimen. As shown in Figure 10a–c, the T8-aged specimen contains uniformly distributed spheroidal δ′ precipitates, whose number density and average size are 3486/μm^2^ and 4.89 nm. As the T8 aging time increases, the number density of T_1_ precipitates is observed to increase progressively, whereas that of δ′ precipitates decreases, accompanied by a concurrent increase in their average diameter. For the T8-100h-aged specimen, the average size of δ′ precipitates increases to 23.58 nm, while their number density decreases to 1981/μm^2^ (Figure 10h). Furthermore, the precipitates around grain boundaries are more uniformly distributed in the T8-aged alloy, and the PFZ is not observed. Compared with the T6-aged specimen, the number density of T_1_ precipitates in the T8-aged alloy increases, together with a higher number density, finer average size, and more uniform distribution of the δ′ precipitates.

### 3.6. Electrochemical Tests

**Figure 11:**
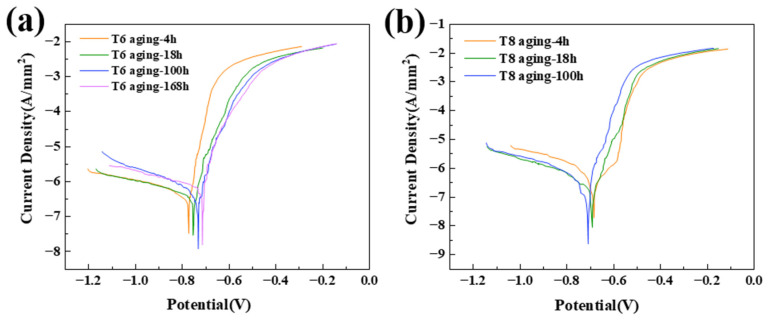
Polarization curves of the T6-aged and T8-aged alloys. (**a**) T6 aging; (**b**) T8 aging.

Figure 11 presents the polarization curves of the T6-aged and T8-aged alloys, and the corresponding electrochemical parameters are listed in Table 5. In electrochemical tests, the corrosion potential (*E_corr_*) and corrosion current density (*I_corr_*) are obtained by fitting the polarization curves. The larger *I_corr_* value and a lower *E_corr_* value indicate higher corrosion susceptibility.

As shown in Table 5, the *E_corr_* and *I_corr_* values of the T6-4 h aged alloy are −0.752 V and 4.562 × 10^−7^ A/cm^2^, respectively; those of the T6-18 h aged alloy are −0.721 V and 3.943 × 10^−7^ A/cm^2^. When the T6 aging time is extended to 168 h, the alloy exhibits the highest *E_corr_* (−0.704 V) and the lowest *I_corr_* (2.384 × 10^−7^ A/cm^2^). These results indicate that the corrosion resistance of the Al-1.1Cu-2.4Li-X alloy improves with increasing T6 aging time. As shown in Figure 11b and Table 5, the *E_corr_* and *I_corr_* values of the T8-4 h aged alloy are −0.667 V and 1.405 × 10^−7^ A/cm^2^, respectively, and those of the T8-100 h aged alloy are −0.709 V and 1.937 × 10^−7^ A/cm^2^. As the T8 aging time increases, the *E_corr_* value decreases, and the *I_corr_* value gradually increases, indicating an increased corrosion susceptibility. Compared with the T6-aged alloy, the T8-aged alloy demonstrates superior corrosion resistance.

## 4. Discussion

### 4.1. Influence of Aging Treatment on Microstructural Evolution

For Al–Li alloys containing an elevated concentration of Li, the high supersaturation of Li atoms contributed to the aging precipitation behavior being sensitive to deformation-induced dislocation structures [9,29]. The above TEM results show that, as the T6 aging time is prolonged, the number density of δ′ precipitates gradually decreases while their diameter increases (Figure 8). For the T8-aged alloys, the evolution of δ′ precipitates is similar to that of the T6-aged alloy, whereas the number density of the T_1_ precipitates increases. Research reveals that the evolution of precipitates is related to the thermodynamics and kinetics [33,34]. During T6 aging, the alloy is directly subjected to artificial aging after solution treatment and quenching [1,12]. The matrix contains supersaturated Li atoms and a high number density of quenched vacancies. The δ′ precipitates are coherent with the matrix, exhibiting an extremely low lattice misfit and nucleation barrier [35,36]. Consequently, homogeneous nucleation occurs at the early aging stage, leading to the dispersed precipitation of a high number density of fine δ′ precipitates. As aging progresses into the over-aging stage, the evolution of the δ′ precipitates is dominated by Ostwald ripening. According to the Lifshitz–Slyozov–Wagner (LSW) theory, the equilibrium Li concentration around fine δ′ precipitates is higher than that around larger ones, which drives solute diffusion from fine δ′ precipitates to large-size particles, causing the dissolution of small particles and the growth of large-size δ′ precipitates [10,16,17]. This process leads to a decrease in the number density of δ′ precipitates and a continuous increase in their average size. Meanwhile, owing to the high Li content, the volume fraction of the δ′ precipitates is relatively high, and the accumulation of coherency strain energy causes large-size δ′ precipitates to gradually become incoherent [26]. Furthermore, during T6 aging treatment, the dislocation density is low, and heterogeneous nucleation sites for the T_1_ precipitates are scarce, which suppresses T_1_ precipitation. As a result, the δ′ precipitates constitute the primary strengthening phase in the T6-aged alloy.

**Figure 12:**
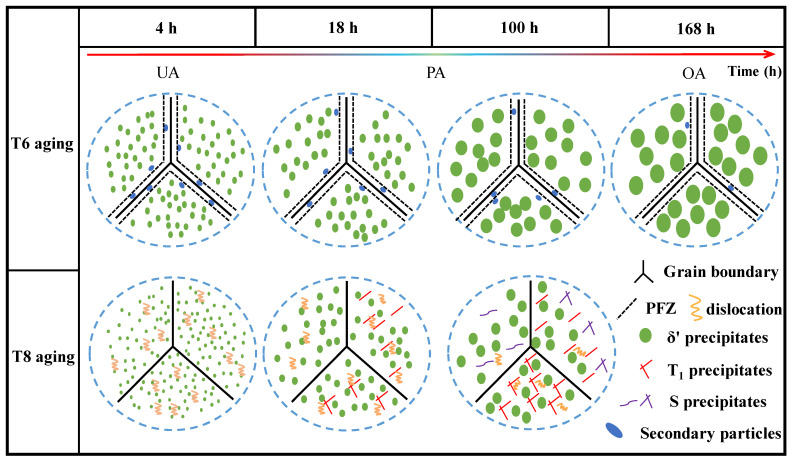
Schematic diagram showing the precipitation behavior of T6-aged and T8-aged samples.

The pre-deformation applied before T8 aging introduces a profuse dislocation substructure into the matrix, thereby modulating the subsequent precipitation sequence. Given that T1 precipitates maintain a semi-coherent interface with the matrix, they exhibit a pronounced preference for preferential nucleation at defect sites (e.g., dislocations and stacking faults) to effectively circumvent the thermodynamic barrier for nucleation [36,37]. The dislocations introduced before T8 aging serve as fast diffusion paths for solute atoms and simultaneously provide abundant preferential nucleation sites for T_1_ precipitates [36,38]. Numerous T_1_ precipitates heterogeneously nucleate on dislocation lines and rapidly grow, depleting Li and Cu atoms from the matrix considerably. This further suppresses the nucleation of δ′ precipitates and promotes the dissolution of fine δ′ precipitates, releasing Li atoms for T_1_ precipitate growth. Furthermore, the T6 and T8 aging temperatures employed in this study are 175 °C and 150 °C, respectively, and the diffusion rate of Li atoms in the matrix is related to temperature. The higher aging temperature applied during T6 aging accelerates Li atom diffusion, which further facilitates the dissolution and coarsening of the δ′ precipitates [12,20,39]. Consequently, the T8-aged alloy, when compared to its T6-aged alloy, displays a greater number density of δ′ precipitates at a finer scale, as well as a pronounced rise in the number density of T_1_ precipitates. As presented in Figure 12, the schematic diagram shows the precipitation behavior of T6-aged and T8-aged samples.

### 4.2. Influence Mechanism of Aging Precipitation Behavior on Mechanical Properties

The mechanical properties of Al–Li alloys are correlated with their precipitation behavior [40,41]. With increasing T6 aging time, the strength gradually increases, which is attributed to the precipitation behavior of δ′ precipitates. Studies show that δ′ precipitates are coherent with the matrix and are more easily sheared by dislocations during plastic deformation. The strengthening contribution can be calculated via the shearing mechanism, as expressed in Equation (1) [42,43]:

(1) $$\Delta \sigma =1.211{\gamma_{eff}}^{\frac{3}{2}}\sqrt{\frac{\pi }{2Gb}}D^{2}N^{\frac{1}{2}}t^{-\frac{3}{2}}$$

where Δ*σ* represents the critical increment in yield strength contributed by the precipitate shearing mechanism; *γ_eff_* is the effective anti-phase boundary (APB) energy when dislocations cut through the precipitates; *G* and *b* are the shear modulus and Burgers vector of the matrix, respectively; *D* is the average particle diameter of the precipitates (e.g., δ′-Al_3_Li); *N* denotes the area number density of the precipitates; and *t* represents the isothermal aging time. According to Equation (1), the contribution of δ′ precipitates to yield strength primarily depends on their average size and volume fraction. With increasing T6 aging time, elemental diffusion within the matrix and nucleation of precipitates become more sufficient. Although some fine δ′ precipitates gradually dissolve, their volume fraction remains essentially stable [44,45,46,47]. Meanwhile, the coarsening of δ′ precipitates is significantly pronounced, which makes the impeding effect of δ′ precipitates on dislocation motion during plastic deformation more complete. Therefore, as the T6 aging time increases, the yield strength and tensile strength increase simultaneously.

Meanwhile, the strength gradually improves as the T8 aging time increases, which is mainly attributed to the precipitation behavior of δ′ and T_1_ precipitates. Research indicates that the plate-like T_1_ precipitates, which possess a hexagonal crystal structure, exhibit an orientation relationship with the Al matrix of {0001}_T1_//{111}_Al_ and <1010> _T1_// <110> _Al_. During the deformation process, T_1_ precipitates exert a greater resistance to dislocation slip along the {111}_Al_ planes, and their strengthening effect is significantly greater compared to that of δ′ precipitates. Moreover, the interaction mechanism between T_1_ precipitates and dislocations is the bypassing mechanism, which can be calculated using the Orowan equation, as shown in Equation (2) [46,47]:

(2) $$\Delta \sigma ({By-passed\;T}_{1})=\frac{0.12Gb}{\sqrt{Dt}}\left(\sqrt{f_{v}}+0.7\sqrt{\frac{D}{t}}f_{v}+0.12\frac{D}{t}{f_{v}}^{\frac{3}{2}}\right)ln\left(\frac{0.079D}{b}\right)$$

In the formula for precipitation strengthening by the T_1_ (Al_2_CuLi) phase, where dislocations bypass the precipitates via the Orowan mechanism, Δ*σ* represents the yield strength increment contributed by this mechanism, *G* and *b* are the shear modulus and Burgers vector of the matrix, respectively, *D* and *t* denote the average diameter and thickness of the disk-shaped T_1_ precipitates, respectively, and f_v_ is the volume fraction of the precipitates. With the prolongation of T8 aging time, the number density of δ′ precipitates decreases while their average size increases. Meanwhile, the number density of T_1_ precipitates increases. These factors collectively contribute to the increase in strength. Furthermore, compared with the T6-aged alloy, the T8-aged alloy contains a higher proportion of δ′ and T_1_ precipitates, which results in significantly higher strength.

### 4.3. Influence Mechanism of Aging Precipitation Behavior on Corrosion Properties

Current research indicates that the intergranular and exfoliation corrosion behavior of specimens subjected to T6 and T8 aging treatments is primarily related to their precipitation characteristics and the precipitation-free zone (PFZ) near the grain boundaries [25,48]. Exfoliation corrosion is essentially a specific manifestation of intergranular corrosion. As corrosion-induced cracks propagate along grain boundaries, the volumetric expansion of corrosion products generates wedging stresses on the surrounding grains, ultimately leading to grain delamination from the surface [49,50,51]. For the T6-aged alloy, in the under-aged condition, the grain interiors are dominated by fine δ′ precipitates and the PFZ exists at the grain boundaries. Moreover, the elements and solute atoms non-uniformly diffuse in the early T6 aging stages, leading to the formation of coarse secondary-phase particles [29,35]. These coarse secondary-phase particles, distributed continuously along the grain boundaries, form micro-galvanic cells with the surrounding matrix: the copper-rich phase has a higher potential and acts as a cathode, accelerating the anodic dissolution of the surrounding Al matrix; the zinc-rich phase has a lower potential and acts as an anode, dissolving preferentially. This localized anodic dissolution process leads to the formation of continuous corrosion pathways along the grain boundaries, thereby significantly aggravating the corrosion. Meanwhile, a significant corrosion potential difference exists between the grain boundary precipitates and the PFZ, with the precipitates acting as the cathode and the PFZ as the anode [51,52,53]. This leads to micro-galvanic corrosion, causing the preferential dissolution of the PFZ and thereby intensifying the susceptibility to both intergranular and exfoliation corrosion. Furthermore, during exfoliation corrosion, the accumulation of corrosion products at grain boundaries induces localized stresses. The PFZ provides space for this accumulation, accelerating the exfoliation process. As the T6 aging time is prolonged, the PFZ width gradually decreases, and a relatively stable passive film tends to form on the surface of the coarsened δ′ precipitates, thereby reducing the number of pitting initiation sites. Thus, as the T6 aging time increases, the corrosion current density of the specimens decreases, and the exfoliation corrosion resistance increases.

For the T8-aged alloy, the fine δ′ precipitates are uniformly dispersed in the early T8 aging stage [54]. Due to dislocations providing abundant heterogeneous nucleation sites, the δ′ precipitates rarely segregate at grain boundaries, thereby suppressing the formation of continuous grain boundary precipitation and PFZ. Meanwhile, the fine δ′ precipitates are smaller than the critical size for passive film breakdown, which contributes to maintaining the integrity of the surface passive film. [36,43] However, as the aging time increases, the T_1_ precipitates gradually increase and coarsen. According to the literature, the anodic dissolution of T_1_ precipitates constitutes the predominant mechanism of intergranular corrosion. Liu et al. [27] demonstrated that the decomposition of Li within the T_1_ precipitates causes their corrosion potential to increase with corrosion time, transforming from anodic to cathodic. Meanwhile, the surrounding matrix and coarse second phases become anodic dissolution zones, which accelerates the corrosion rate. In addition, corrosion tends to initiate preferentially in regions with a high concentration of T1 precipitates and propagate along dislocations, resulting in pitting of T_1_ precipitates and sub-grain boundary corrosion [55,56]. Therefore, as T8 aging progresses, both the self-corrosion current density and the exfoliation corrosion rate of the specimen increase simultaneously. The *E_corr_* value of T8-100 h is lower than that of T8-4 h because pre-straining promotes the extensive precipitation of the T_1_ phase, resulting in a negative shift in the alloy’s open-circuit potential; however, its corrosion resistance remains superior to that of the T6 condition. The fundamental reason lies in the uniform and dense precipitation of the T_1_ phase within the grains in the T8-aged condition, which effectively reduces the potential difference between the grain interior and the grain boundaries, thereby lowering the driving force for intergranular corrosion. At the same time, the discontinuous distribution of precipitation phases at the grain boundaries interrupts continuous corrosion pathways, and pre-straining effectively suppresses the formation of the PFZ. However, over-aging inevitably leads to the growth of intergranular precipitates and the coarsening of the T_1_ phase, resulting in increased susceptibility to intergranular corrosion; therefore, the reason why the T8-100 h condition still exhibits superior corrosion resistance to the T6 condition is that, although the coarsening of the T_1_ phase and the presence of the PFZ in the T8-100 h condition exist, the adverse effects of the PFZ are not ‘eliminated’, but rather, by altering the local electrochemical environment, corrosion is shifted from a grain-boundary propagation mode to a mode dominated by pitting corrosion. The damage caused by pitting corrosion is generally less severe than that caused by intergranular corrosion and exfoliation corrosion; consequently, in an overall assessment of corrosion resistance, the T8-100 h condition remains superior to the T6 condition. Meanwhile, the insoluble coarse phases distributed at grain boundaries during the homogenization treatment are broken up during the pre-deformation treatment prior to T8 aging. Thus, compared with the T6-aged alloy, the T8-aged alloy exhibits the disappearance of PFZs, a reduction in coarse secondary-phase particles around the grain boundaries, and a more uniform distribution of the δ′ phase within the matrix. These factors collectively result in T8-aged alloys showing enhanced corrosion resistance.

## 5. Conclusions

In the present study, the corrosion behavior (specifically encompassing intergranular and exfoliation corrosion) and mechanical properties of T6- and T8-aged Al–Li alloys are systematically investigated. The principal conclusions are outlined as follows:

(1) With the prolongation of the T6 and T8 aging times, the yield strength and tensile strength gradually increase. The yield strength and tensile strength of T6-168 h samples are 374.4 MPa and 469.9 MPa. Additionally, the yield strength and tensile strength of T8-aged alloy are 90 MPa and 40 MPa higher, respectively, than those of T6-aged alloy.

(2) The T6-aged alloy is primarily strengthened by δ′ precipitates. With prolonged T6 aging, the number density of δ′ precipitates decreases, their diameter increases, and the width of the precipitate-free zone (PFZ) diminishes. In contrast, the T8-aged alloy contains both δ′ and T_1_ precipitates as its main strengthening phases. In comparison with the T6-aged condition, the T8-aged alloy exhibits a higher number density of T_1_ precipitates, along with a higher number density of δ′ precipitates that are finer and more uniformly dispersed.

(3) The intergranular corrosion depths of the T6-aged and T8-aged alloy specimens range from 176.5 μm to 210.1 μm and 67.2 μm to 95.8 μm, respectively. Compared with T6-aged specimens, the T8-aged counterparts demonstrate enhanced resistance against intergranular and exfoliation corrosion.

(4) The *E_corr_* and *I_corr_* values of the T6-4 h-aged alloy are −0.752 V and 4.562 × 10^−7^ A/cm^2^, while for the T6-168 h-aged alloy, the *E_corr_* increases to −0.704 V and the *I_corr_* decreases to 2.384 × 10^−7^ A/cm^2^. The corrosion resistance is improved with increasing T6 aging time, whereas the *E_corr_* value decreases and the *I_corr_* value gradually increases as the T8 aging time increases, indicating increased corrosion susceptibility.

(5) Based on the trade-off between mechanical properties and corrosion resistance in high-Li Al–Li alloys, the recommended applications are as follows:

T8-4 h provides high strength combined with excellent intergranular and pitting corrosion resistance, and is recommended for load-bearing structures in corrosive environments.

T8-168 h reaches peak strength but shows higher corrosion susceptibility; it is suited for high-strength parts in mild environments when surface protection is applied.

T6-168 h gives the lowest strength with the best corrosion resistance, and can serve as an alternative for applications such as storage-tank inner walls.

Overall, short-term T8 aging (4 h) delivers the best balance of properties and is the preferred choice.

## Figures and Tables

**Table 1:** Chemical compositions of the experimental alloy (wt.%).

Alloy	Cu	Li	Mg	Zn	Mn	Zr	Al
Al-1.1Cu-2.4Li-X	1.08	2.44	0.46	0.32	0.34	0.12	95.24

**Table 2:** Mechanical properties of the alloys subjected to T6 and T8 aging treatments.

Aging Treatment		Tensile Strength (MPa)	Yield Strength (MPa)	Elongation
T6 aging	4 h	414.9	292.8	12.4
	32 h	451.4	350.0	10.1
	72 h	469.6	366.7	8.9
	100 h	469.6	370.4	8.7
	168 h	469.9	374.4	9.4
T8 aging	4 h	377.0	255.6	21.2
	32 h	460.2	412.0	11.4
	72 h	497.6	442.6	8.2
	100 h	506.7	447.2	8.6
	168 h	512.0	463.0	8.9

**Table 3:** Types of intergranular corrosion exhibited by the Al-1.1Cu-2.4Li-X alloy.

Types of Corrosion	Features	Criteria
Generalized intergranular corrosion	Morphology of lattice-like intergranular corrosion	More than 80% of the total observed area
Localized intergranular corrosion	Morphology of lattice-like intergranular corrosion	Less than 80% of the total observed area
Pitting corrosion	Tiny pitting	Erosion pits of varying sizes, with a non-grid-like pattern
Pitting corrosion accompanied by intergranular corrosion	Slight intergranular corrosion has appeared around the base of the corrosion pit	The depth of the pitting is considerably greater than that of the accompanying microcracks

**Table 4:** Exfoliation corrosion evaluation level for T6-aged and T8-aged alloys.

Corrosion Time	T6 Aging Time			T8 Aging Time	
	4 h	100 h	168 h	4 h	100 h
4 h	N	N	N	N	N
24 h	PB	PA	PA	PA	PB
48 h	PC	PB	PB	PA	PC
72 h	EC	EB	EA	PC	EB
96 h	ED	EC	EA	PC	EC

**Table 5:** Electrochemical parameters of the T6-aged and T8-aged samples.

Aging Treatment		OCP (V)	*E_corr_* (V)	*I_corr_* (A/cm^2^)	R_ct_ (Ω/cm^2^)
T6 aging	4 h	−0.786	−0.752	4.562 × 10^−7^	2279
	18 h	−0.764	−0.721	3.943 × 10^−7^	2635
	100 h	−0.741	−0.736	2.792 × 10^−7^	3280
	168 h	−0.729	−0.704	2.384 × 10^−7^	4306
T8 aging	4 h	−0.709	−0.667	1.405 × 10^−7^	4925
	18 h	−0.714	−0.683	1.643 × 10^−7^	4261
	100 h	−0.735	−0.709	1.937 × 10^−7^	3882

## Data Availability

The raw data supporting the conclusions of this article will be made available by the authors on request.
